# Genetic mechanism regulating diversity in the placement of eyes on the head of animals

**DOI:** 10.1073/pnas.2316244121

**Published:** 2024-04-08

**Authors:** Oorvashi Roy Puli, Neha Gogia, Anuradha Venkatakrishnan Chimata, Takeshi Yorimitsu, Hideki Nakagoshi, Madhuri Kango-Singh, Amit Singh

**Affiliations:** ^a^Department of Biology, University of Dayton, Dayton, OH 45469; ^b^Graduate School of Environmental, Life, Natural Science and Technology, Okayama University, Okayama 700-8530, Japan; ^c^Premedical Program, University of Dayton, Dayton, OH 45469; ^d^Integrative Science and Engineering, University of Dayton, Dayton, OH 45469; ^e^Center for Genomic Advocacy (TCGA), Indiana State University, Terre Haute, IN 47809

**Keywords:** *Drosophila* eye, retinal determination, defective proventriculus, SATB1, Wingless

## Abstract

Despite the conservation of genetic machinery involved in eye development, there is a strong diversity in the placement of eyes on the head of animals. Morphogen gradients of signaling molecules are vital to patterning cues. During *Drosophila* eye development, Wingless (Wg), a ligand of Wnt/Wg signaling, is expressed anterolaterally to form a morphogen gradient to determine the eye- versus head-specific cell fate. The underlying mechanisms that regulate this process are yet to be fully understood. We characterized *defective proventriculus (dve)* (*Drosophila* ortholog of human SATB1), a K50 homeodomain transcription factor, as a dorsal eye gene, which regulates Wg signaling to determine eye versus head fate. Across *Drosophila* species, Dve is expressed in the dorsal head vertex region where it regulates *wg* transcription. Second, Dve suppresses eye fate by down-regulating retinal determination genes. Third, the *dve*-expressing dorsal head vertex region is important for Wg-mediated inhibition of retinal cell fate, as eliminating the Dve-expressing cells or preventing Wg transport from these *dve*-expressing cells leads to a dramatic expansion of the eye field. Together, these findings suggest that Dve regulates Wg expression in the dorsal head vertex, which is critical for determining eye versus head fate. Gain-of-function of SATB1 exhibits an eye fate suppression phenotype similar to Dve. Our data demonstrate a conserved role for Dve/SATB1 in the positioning of eyes on the head and the interocular distance by regulating Wg. This study provides evidence that dysregulation of the Wg morphogen gradient results in developmental defects such as hypertelorism in humans where disproportionate interocular distance and facial anomalies are reported.

## Significance

Several studies highlight the role of morphogens during craniofacial development. We identified a conserved role for Defective proventriculus, Dve, the *Drosophila* ortholog of human SATB1, in the positioning of eyes on the head and the interocular distance by regulating Wg (Wingless) transcription and its gradient. Deregulation of the Wg morphogen gradient results in developmental defects like craniofacial abnormalities including hypertelorism in humans. However, the genetic basis of how it is caused is not clear. This study shows that a transcription factor, Dve, has a conserved function in regulating the transcription of Wg and its gradient from a subset of cells in the developing eye–antennal imaginal disc to determine the placement of eyes on the head.

During organogenesis, events like cell fate specification and patterning decisions are dependent on an optimal balance of evolutionarily conserved signaling pathways. Signaling molecules and their long/short-range distribution affect the cell fate decisions in developing fields. In all multicellular organisms, the cells within the field interpret their position and function with respect to a concentration or gradient of signaling molecules (like morphogens) to determine their developmental fate(s).

Wingless (Wg), a long-range secretory molecule of the Wnt family, generates a morphogen gradient to regulate developmental processes (1). This occurs as 1) “Wnt- or Wg-producing cells” produce and secrete the protein for paracrine signaling, 2) the Wg morphogen is transported to receiving cells and spreads over several cells through the tissue to form a gradient, and 3) these cells respond to Wg in a concentration-dependent manner to activate target genes that regulate pattern formation and determine cell identities (2). The Wg ligand can act as both a short- and long-range signaling molecule to organize or orchestrate the expression of other genes crucial for development. Restricting autonomous, short-, or long-range Wg signaling impacts the expression of its target gene *bifid* (*bi*) differently in the eye (3). In the *Drosophila* eye, the level and distribution of Wg are crucial for various functions such as growth, cell proliferation, patterning, cell death, and suppression of eye fate (4). In humans, dysregulation of morphogen levels affects growth and patterning and results in developmental birth defects (5, 6).

The *Drosophila* eye develops from larval eye imaginal discs. The early eye primordium undergoes transition from a monolayer epithelium to a three-dimensional organ by delineation of anterior–posterior (AP), dorsal–ventral (DV), and proximal–distal (PD) axes (7). In *Drosophila*, the eye fate is specified by the expression of *eyeless* (*ey*) in the entire eye primordium. The DV axis is the first lineage restriction event, which results in formation of an equator at the boundary of dorsal and ventral compartments. Dorsal eye fate is established by expression of the GATA-1 transcription factor *pannier (pnr)*, which regulates *wg* and homeodomain genes of the Iroquois complex (7–10). In the *Drosophila* eye disc, Wg is expressed at the anterolateral margin.Wg negatively regulates photoreceptor differentiation and determines the eye versus head fate (11–14). Loss-of-function (LOF) of *wg* in the developing eye results in eye enlargements both on dorsal and ventral margins due to generation of ectopic morphogenetic furrows (MF) (13, 14). MF marks a synchronous wave of retinal differentiation in the developing eye (15). In the dorsal eye, spatiotemporal regulation of *wg* is complex. We have previously shown that Pnr also regulates Wg, although it may not be the direct and sole regulator of Wg in the developing eye (10).

**Fig. 1.**
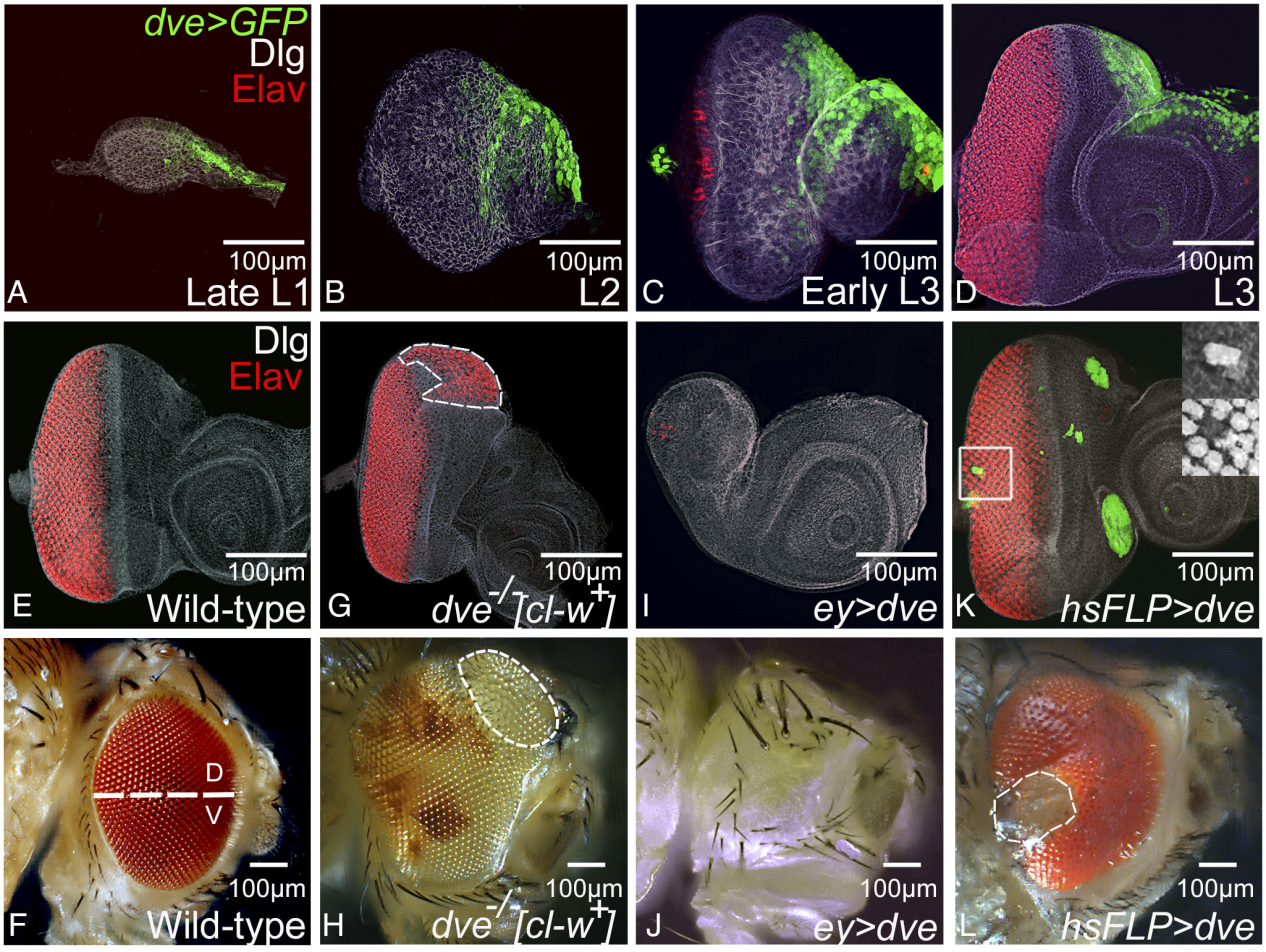
*dve*, a DV patterning gene is expressed in the dorsal head vertex region of the developing eye. (*A*–*D*) The spatiotemporal expression profile of *dve* was detected using *dve* Gal4 > UAS-GFP (green) in the developing (*A*) late L1, (*B*) L2, (*C*) early L3, and (*D*) L3 larval eye imaginal disc. *dve* expression (green) in the eye–antennal imaginal disc of the (*A*) late first-instar larva (L1) is initiated in 15 to 20 cells, close to the anterior margin. Note that discs are stained with Disc large (Dlg), a membrane-specific marker (white), and the panneural marker Elav (red) that marks the photoreceptor neurons. (*B*) In the early second-instar larva (L2), expression is restricted to a group of 30 to 40 cells on the dorsal eye margin, and in (*C*) early third-instar larva (early L3), expression is seen in the antenna and dorsal head vertex region. Note that the retinal differentiation has just initiated at this stage as seen by a single row of Elav-positive cells on the posterior margin. (*D*) In the late third-instar larva (late L3), expression is restricted to a group of cells on the dorsal eye margin and the antenna region. Note that *dve* expression is anterior to the furrow and does not overlap with the differentiated photoreceptor neurons on the dorsal margin. (*E*) Wild-type third-instar eye imaginal disc, which develops into a (*F*) compound adult eye. (*G* and *H*) LOF of *dve*, using a cell-lethal approach (20), results in dramatic dorsal eye enlargements as seen in the (*G*) eye imaginal disc and (*H*) adult eye. Note that loss of *dve* in the ventral eye has no effect. The white-dotted line marks the outline of enlargements in the eye imaginal disc and the adult eye. In the adult eye, LOF clones are marked by the absence of mini-*white* reporter. (*I* and *J*) Misexpression of *dve* (*ey* > *dve*) in the *ey* driver domain of the entire eye imaginal disc using *ey*-Gal4 driver results in suppression of the eye as evident from loss of Elav (red) expression in the (*I*) eye imaginal disc and the (*J*) adult eye. Adults of *ey* > *dve* showed no-eye. (*K* and *L*) Heat shock flippase–mediated generation of random GOF clones of *dve* marked by GFP reporter showed suppression of panneural marker Elav expression in the eye imaginal disc. All images/panels will have dorsal (D) up and ventral (V) down.

We have identified *defective proventriculus (dve)* as a DV patterning gene that regulates *wg* expression in the developing eye. Dve encodes a K50 homeodomain transcription factor, an ortholog of human SATB1 (16, 17), which is required for regulation of gene expression in retinal ganglion cells (18). In the late first-instar eye disc, *dve* expression begins in a small subgroup of cells (Fig. 1*A*). Expression of *dve* remains restricted to the anterodorsal eye disc near the head vertex region in the late second- and early third-instar stages (Fig. 1 *B*–*D*) and evolves to a small subset of ~150 to 200 dorsal cells in the third-instar eye imaginal disc as detected by the *dve-*reporter as well as Dve antibody (*SI Appendix*, Fig. S2). In the developing eye, LOF of *dve* results in dorsal eye enlargements along with the loss of ocelli (Fig. 1 *G* and *H* and *SI Appendix*, Fig. S1), whereas gain-of-function (GOF) of *dve* results in reduced eye size (Fig. 1 *I* and *J*). Random “flp-out” GOF (19) clones of *dve* also suppressed eye fate irrespective of the clone position in the dorsal or ventral domain of the developing eye (Fig. 1 *K* and *L*). This suggests that *dve* suppresses eye development. Therefore, we investigated the effect(s) of *dve* misexpression on retinal determination (RD) genes.

**Fig. 2.**
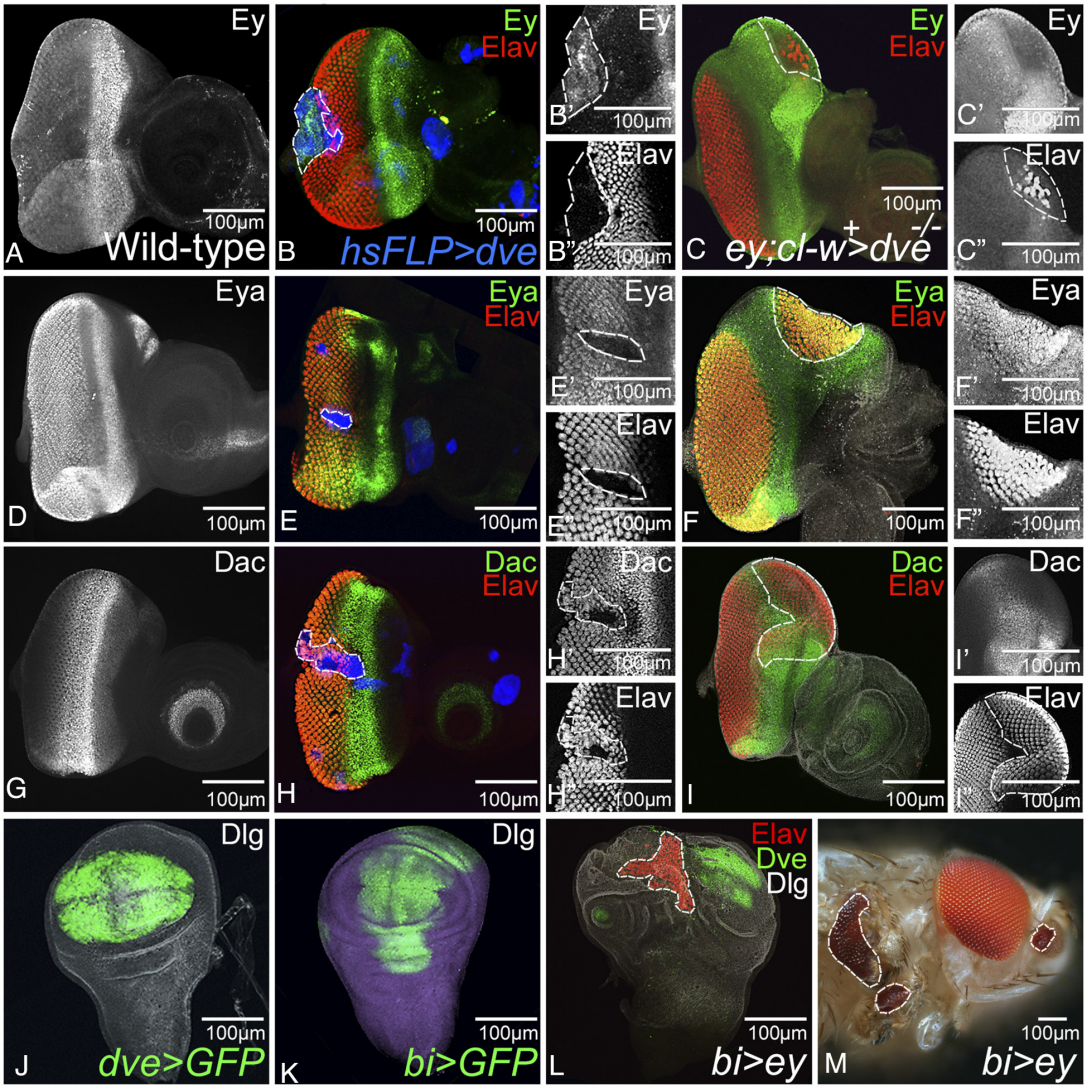
*dve* suppresses eye fate by down-regulating expression of the highly conserved RD gene network. (*A*) Wild-type expression of Ey in the anterior region of the third-instar eye disc. (*B*) Random GOF clones of *dve* marked by GFP (blue) exhibit induction of (*B*, and *B’*) Ey (green) whereas LOF clones of *dve* generated by the *cell-lethal* approach result in downregulation of (*C*, and *C’*) Ey. Note that Ey is an eye field specification marker which acts upstream of RD genes *eya* and *dac*, and differentiated photoreceptor neurons are marked by ELAV (red). (*D*) Wild-type expression of Eya, a RD gene, is seen both anterior and posterior to the MF and in the ocelli region of eye disc. (*E*–*E”*) GOF clones of *dve* marked by GFP (blue) exhibit suppression of (*E*, *E’*) Eya (green) and suppression of eye fate marked by (*E*, *E”*) ELAV (red). (*F*) LOF clones of *dve* result in ectopic eye formation along with induction of (*F* and *F’*) Eya (green) and Elav (red). (*G*) Dac expresses posterior to the MF in R1, R6, and R7 photoreceptor as well as anterior to the MF in a narrow band of cells of the wild-type eye imaginal disc. (*H*–*H”*) Random GOF clones of *dve* marked by GFP (blue) exhibit suppression of (*H* and *H’*) Dac (green) and ectopic induction of (*H* and *H”*) Elav (red) expression. (*I*–*I”*) LOF clones of *dve* results in downregulation of (*I* and *I’*) Dac along with dorsal eye enlargement as evident from (*I”*) ectopic Elav expression. (*J* and *K*) Expression domains of Gal4 drivers in the wing-imaginal discs. (*J*) *dve*-Gal4 (*dve* > GFP) and (*K*) *bi*-Gal4 (*bi* > GFP) drive GFP reporter expression (green) in the wing pouch. (*L* and *M*) Misexpression of *ey* in the *bi*-Gal4 domain induces an ectopic eye in the (*L*) larval wing-imaginal disc, based on ectopic Elav (red) expression, and (*M*) wing, leg, and antenna of the adult fly. (*L*) Note that Dve (green) expression is down-regulated in the wing pouch where ectopic *ey* induces an ectopic eye.

A cascade of highly conserved genes like *ey, eyes absent (eya), sine oculis (so),* and *dachshund (dac)* are required for eye field specification and differentiation (21). The eye suppression phenotype of *dve* GOF results in ectopic expression of the eye specification marker Ey (Fig. 2 *B* and *B’*) and suppression of retinal differentiation markers, Eya (Fig. 2 *E* and *E’*) and Dac (Fig. 2 *H* and *H’*). However, dorsal eye enlargement phenotypes of *dve* LOF clones showed no effect on Ey expression (Fig. 2 *C* and *C’*) but showed ectopic induction of downstream markers, Eya (Fig. 2 *F* and *F’*) and Dac (Fig. 2 *I* and *I’*). Our data suggest that *dve* may not affect eye specification but regulates retinal differentiation. We tested whether *dve* function is required to regulate differentiation by using an ectopic eye assay. Ectopic induction of *ey* in the wing pouch results in ectopic eyes in the wing disc (22) (Fig. 2 *L* and *M*). We used an *optomotor blind (omb)* or *bi*-Gal4 driver (12) to target expression of the *ey* transgene in the wing pouch (Fig. 2 *K* and *L*). Ectopic expression of master eye fate regulator, *ey*, induces retinal differentiation in the wing by repressing Dve expression in the wing pouch region (Fig. 2 *J* and *L*), suggesting that *dve* acts as an antagonist of RD gene function.

**Fig. 3.**
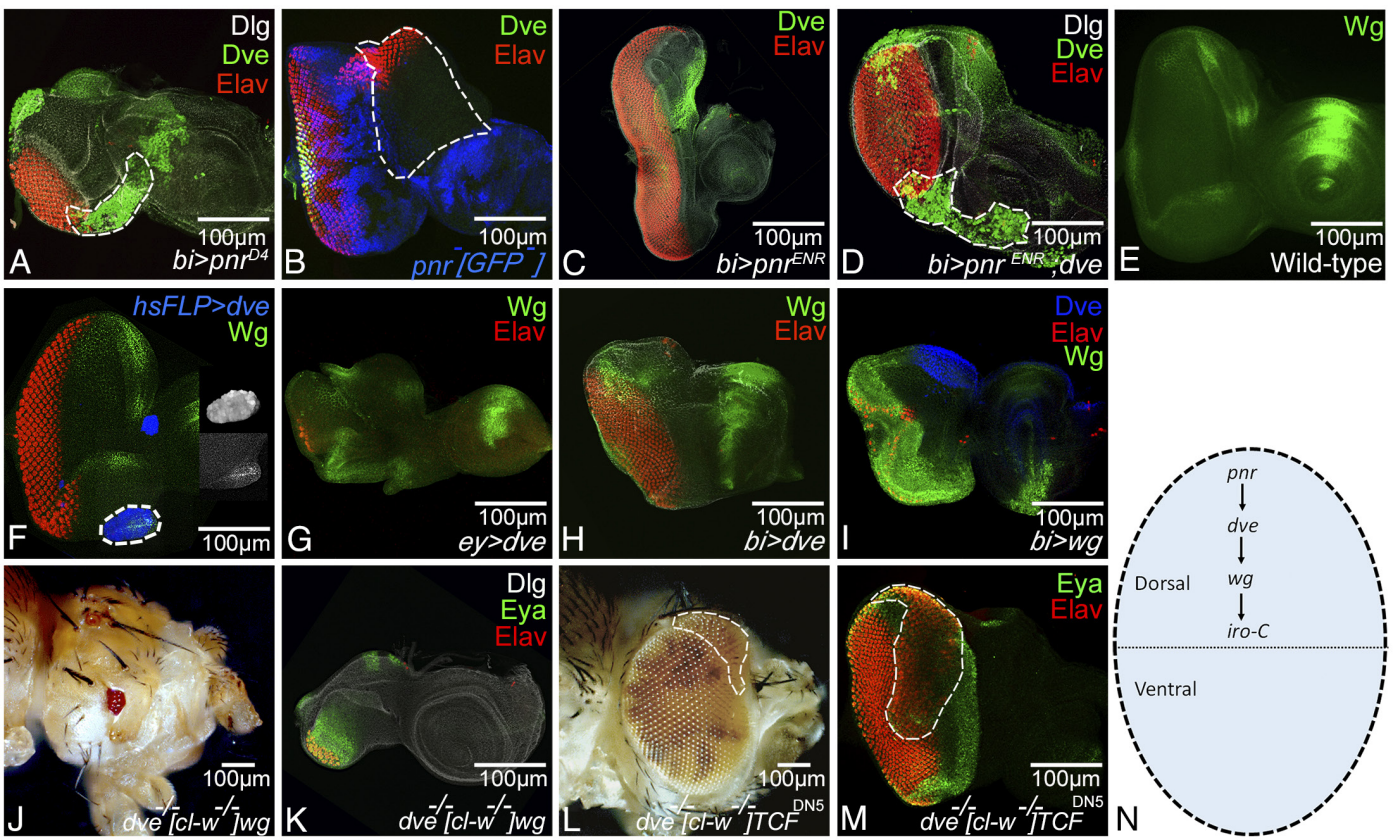
In dorsal eye gene hierarchy, *dve* acts downstream of *pnr* and upstream of *wg*. (*A*) Misexpression of *pnr* using the *bi-*Gal4 driver *(bi* > *pnr^D4^)* causes ectopic induction of Dve (green) in the ventral eye (marked by the white-dotted line). The endogenous expression of Dve in the dorsal eye can be seen along with *pnr-*mediated ectopic Dve expression on the ventral margin. (*B*) Loss of function of *pnr* marked by the absence of GFP (blue; white-dotted line marks the boundary of the clone) shows ectopic eye formation as seen by ectopic Elav (red) and loss of endogenous Dve (green) expression. (*C*) Loss of *pnr* on DV eye imaginal disc margin by using dominant negative *pnr* (*bi* > *pnr^ENR^*) causes dorsal eye enlargements, which can be suppressed by (*D*) coexpressing *dve* (*bi* > *pnr^ENR^*+*dve*) on the DV margins suggesting that *dve* GOF rescues the *pnr* LOF phenotype. Therefore, *dve* acts downstream of *pnr*. (*E*) Wild-type Wg expression (green) is restricted to the anterolateral margins of the developing eye imaginal disc. (*F*) Random GOF clones of *dve* marked by GFP (blue) in the eye disc cause ectopic induction of Wg (green). The inset shows the magnified view of the clone. (*G* and *H*) Misexpression of *dve* (*G*) in the entire developing eye using an *ey-*Gal4 (*ey* > *dve*) and (*H*) on the DV margins using *bi-*Gal4 resulted in upregulation of the Wg protein level (green) and suppression of eye fate as seen by Elav (red) expression. (*I*) Misexpression of *wg* on DV margins using *bi*-Gal4 (*bi* > *wg*) did not induce Dve, suggesting that the *dve* gene acts upstream of *wg*. (*J*–*M*) In *dve* LOF clones, generated by a cell-lethal approach, modulating levels of Wg signaling by (*J* and *K*) misexpressing Wg in the eye results in highly reduced eye, whereas (*L* and *M*) misexpressing dTCF^DN5^, a dominant negative construct of TCF, results in ectopic dorsal eye enlargements. (*N*) Schematic representation of genes in the dorsal eye gene hierarchy.

LOF and GOF phenotypes of *dve* in the eye are similar to another DV patterning gene, *pnr* (Fig. 1 and *SI Appendix*, Fig. S1) (10). Even though their expression has partial overlap in the dorsal eye, *dve* has distinctively unique expression domain in the dorsal eye. The dorsal eye gene *pnr* is expressed in the dorsal eye margin as well as the peripodial membrane, whereas Dve is expressed in the disc proper in the dorsal head vertex region (*SI Appendix*, Fig. S3). Our genetic epistasis analysis assigns *dve*, *wg,* and other dorsal genes to the dorsal gene hierarchy on the basis of expression and phenotypes (Fig. 1). We used *bi*-Gal4 to drive expression of transgenes on DV margins of the developing eye (12) (*SI Appendix*, Fig. S4). *bi*-Gal4 can serve as an excellent tool for genetic epistasis experiments for dorsal eye genes as it drives expression both on the dorsal as well as ventral margins of the developing eye. Misexpression of *pnr* on the DV margin (*bi* > *pnr*) of the eye ectopically induces Dve on the ventral margin (Fig. 3*A*). LOF clones of *pnr* in the dorsal eye exhibit complete loss of *dve* expression (Fig. 3*B*). Additionally, the dorsal eye enlargement phenotype of dominant-negative *pnr* (*pnr^ENR^*) misexpression (*bi* > *pnr^ENR^*, Fig. 3*C*) is rescued when *dve* is coexpressed with *pnr^ENR^* (*bi* > *dve* +*pnr^ENR^*, Fig. 3*D*). Thus, GOF of *dve* masks the phenotype of LOF of upstream *pnr,* thereby suggesting that *dve* acts downstream of *pnr.* Ectopic flp-out clones of *dve* induce Wg in the eye disc (*hsFLP* > *dve*, Fig. 3*F*), and misexpression of *dve* in the posterior eye (*ey* > *dve*, Fig. 3*G*) or on the DV boundary (*bi* > *dve*, Fig. 3*H*) can induce ectopic Wg expression and thereby restrict retinal differentiation. Modulation of Wg levels in a *dve* mutant background can suppress (Fig. 3 *J* and *K*) or enhance (Fig. 3 *L* and *M*) the *dve* mutant phenotype of dorsal eye enlargement. The reduced eye phenotype of GOF of *wg* can mask the dorsal eye enlargement phenotype of *dve* LOF suggests that *dve* acts upstream of *wg* in the dorsal eye (Fig. 3*N*).

**Fig. 4.**
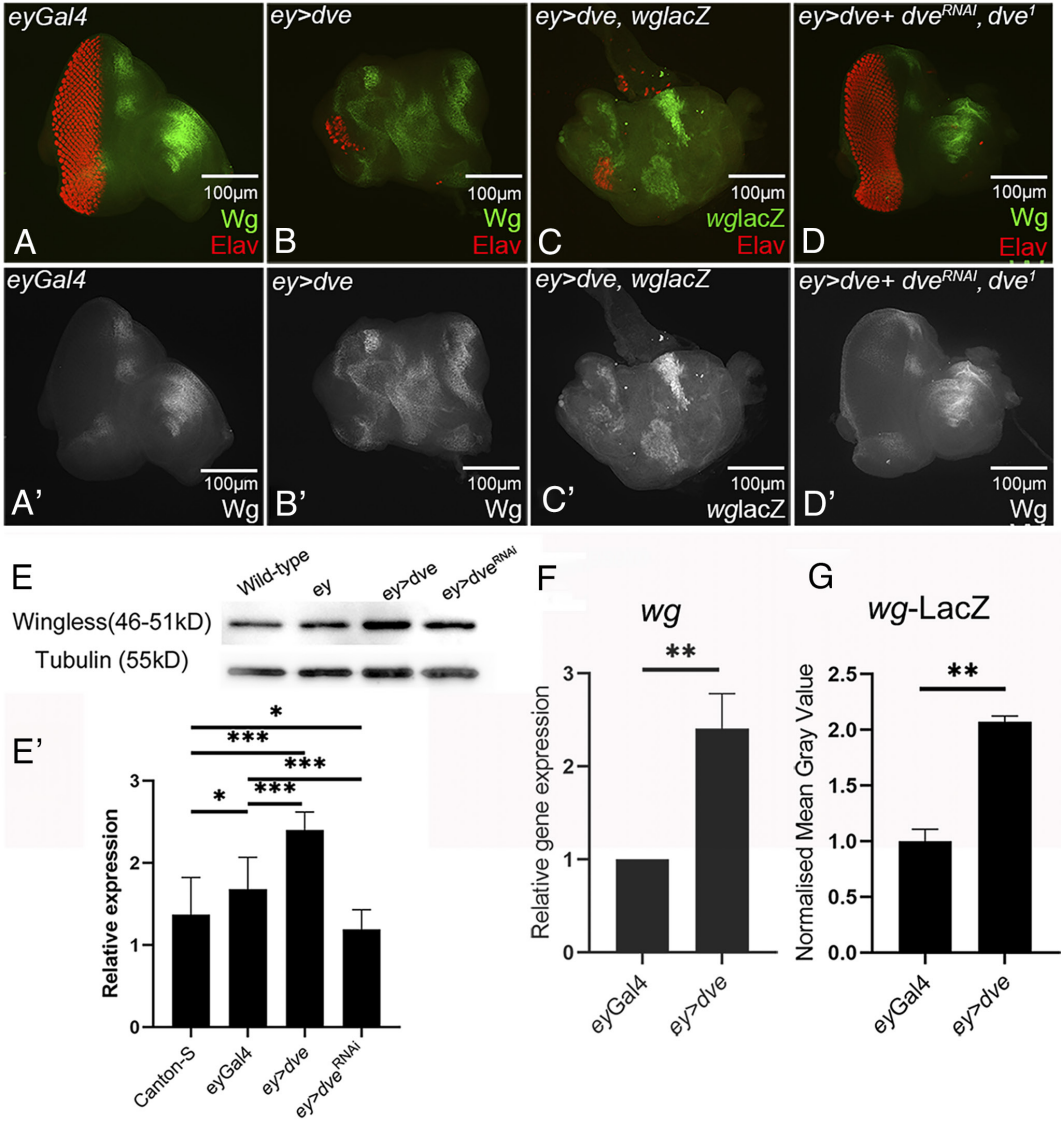
Dve induces Wg expression. (*A* and *A’*) Wild-type Wg expression in *eyGAL4* control eye discs. (Fig. 4 *B* and *B’*) GOF of *dve* in the developing eye, using *ey*-Gal4 driver (*ey* > *dve*) results in upregulation of Wg (green) expression in the posterior margin of the eye disc. (*C* and *C’*) In addition, the transcriptional readout of the wg gene using lacZ reporter (green) showed ectopic induction in the posterior eye (*C’*) resulting in the reduced eye marked by panneural marker Elav (red) expression. (*D* and *D’*) LOF of *dve* by misexpressing *dve ^RNAi^* in the developing eye, using *ey*-Gal4 (*ey* > *UAS dve^IR^, dve^[1]^*), *ey* domain shows (*D*) normal eye and head formation as evident from Elav (red) and (*D’*) Wg expression comparable to the wild type. (*A’*, *B’*, *C’*, and *D’*) show the individual Wg expression channel. (*E* and *E’*) Western blot image and analysis (*E*) blot shows Wg and tubulin levels in control groups, GOF and LOF of dve. (*E’*) Semiquantitative analysis shows changes in Wg levels. (*F*) Real-time qPCR analysis shows significant upregulation of *wg* upon misexpression of *dve (ey* > *dve)* when compared to *ey*-Gal4 control. (*G*) Quantitative analyses of *wg* levels (*wg*-LacZ) in the eye discs (n = 5, 20×) are represented as normalized mean gray values and show significant difference between *dve* GOF *(ey* > *dve)* and control (*ey*-Gal4) groups.

Using a transcriptional readout of *wg* (*wg-lacZ* reporter) (23), misexpression of *dve* in the posterior eye showed (fold change:~2 time, *P*-value: 0.002) ectopic *wg-*lacZ induction (*ey* > *dve*, Fig. 4 *C*–*C”* and *G*). It suggests that *wg* is a transcriptional target of Dve. GOF of *dve* (*ey* > *dve*) in the eye exhibits ectopic Wg induction (Fig. 4 *B* and *B'*). LOF of *dve* by the RNAi approach restored the “no-eye” phenotype of *ey* > *dve* +*dve^RNAi^* (Fig. 4*D*). Real-time quantitative PCR analysis revealed significant upregulation in *wg* mRNA (fold change: ~2.4, *P*-value: 0.009) (Fig. 4*F*). Furthermore, semiquantitative western blot analysis exhibits a ~two fold increase in Wg protein levels, respectively (Fig. 4 *E* and *E’*). Thus, our data suggest that during eye development, the DV patterning gene, *dve* acts downstream of *pnr* and upstream of *wg* and regulates *wg* expression (Figs. 3 and 4). Earlier, we have shown that Dve is also required for specification of the ocellar region (24). Dve expression in the early third larval instar depends on Orthodenticle (Otd) (24). However, *otd* mutation does not induce eye enlargement phenotype(s). This difference might be due to the Otd-independent and Pnr-dependent Dve expression induced in late first larval instar (Fig. 1*A*).

**Fig. 5.**
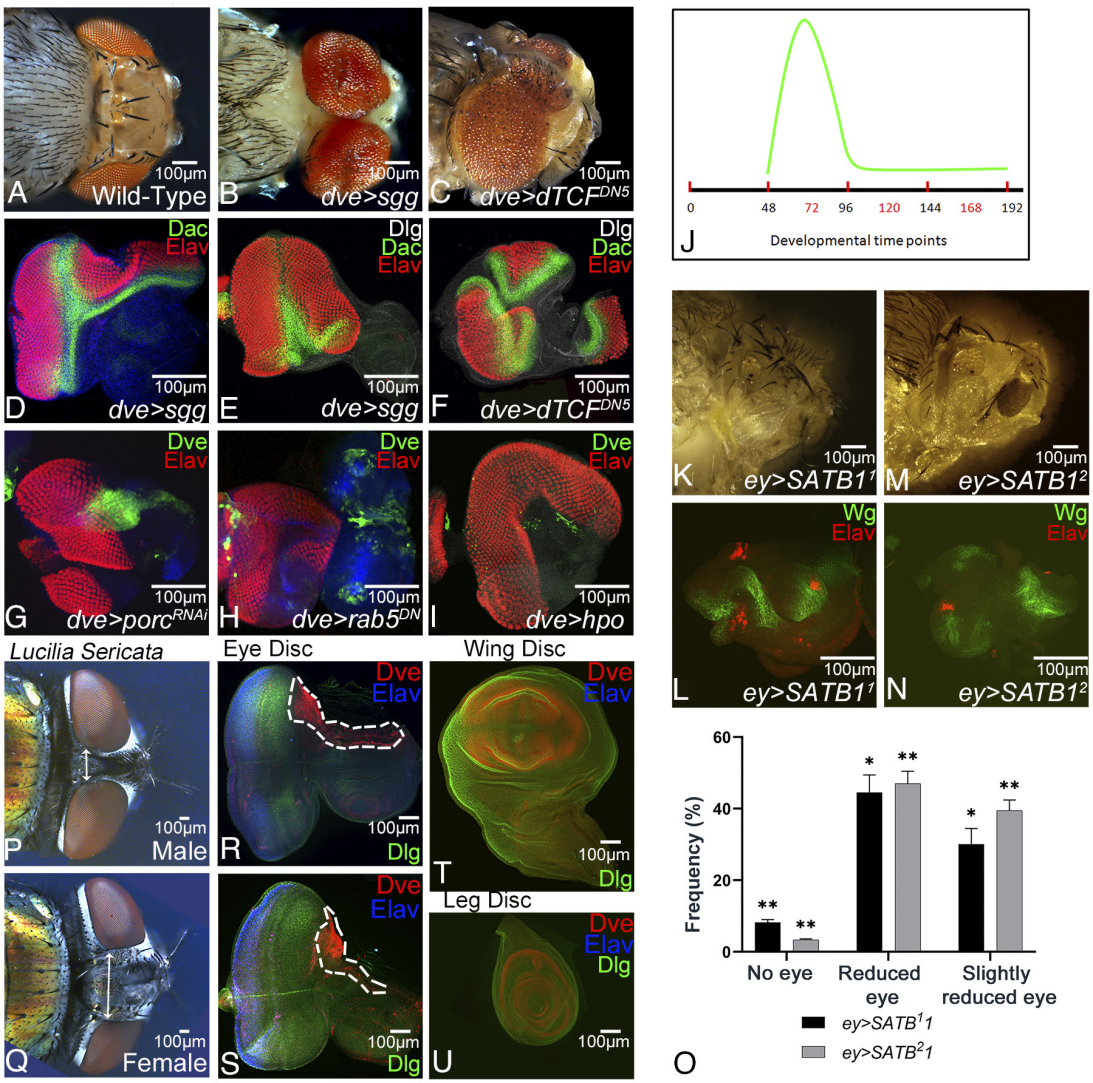
*dve* determines eye versus head fate through a conserved mechanism. (*A*–*C*) Dorsal view of adult head from (*A*) wild-type showing the ocellar region and the spacing of two eyes is compared to adult heads from (*B*) *dve*>*sgg* and (*C*) *dve*>*dTCF^DN^* in which Wg signaling is blocked. (*D*–*F*) Panels show eye discs in which misexpression of (*D*, *E*) Shaggy-kinase (*dve*>*sgg*) results in the transformation of head cuticle to eye fate. A comparison of *D* and *E* shows the range of phenotypes where in (*D*) ectopic eye enlargements in the *dve* domain results in the loss of head cuticle on the dorsal margin in the eye disc whereas in (*E*) a de novo eye field is generated in the anterior portion of the eye disc which results in a duplicated furrow marked by Dac (green) and duplication of the eye field as seen by Elav expression (red). In such discs the entire head forming region of eye disc changes to eye specific fate. (*F*) Eye disc showing misexpression of dominant negative TCF (*dve*>*dTCF^DN^*) also results in transformation of dorsal head cuticle and antenna to an eye fate and formation of de novo eye field as seen by expression of ELAV (red) that marks differentiated photoreceptor neurons and Dac (green) that marks the MF in the eye disc. (*G*, *H*) Blocking Wg transport from the *dve* expressing cells by (*G*) misexpressing *porcupine-RNAi* (*dve*>*porc^RNAi^*) and (*H*) a dominant negative form of *rab5* (*dve*>*rab5^DN^*) shows similar eye enlargement (*I*) Eliminating *dve* expressing cells by misexpression of Hippo (*dve*>*GFP+hpo*) also resulted in huge enlargements of the eye field. Note that absence of GFP (green) reports the loss of *dve* expressing cells. (*J*) Using temperature sensitive *Gal80^ts^* allele, Gal4 mediated misexpression of UAS-transgene can be controlled. The graph displays the crucial developmental time point (~48-96 hours AEL) when *dve* expressing cells are required to delineate the eye and head fate. (*K*–*O*) SATB1, a human ortholog of Dve when misexpresssed in the developing eye imaginal disc using two independent SATB1 transgenes (*ey>SATB^1^1 ey>SATB^2^1*) suppresses (*K*, *M*) the eye formation in adults, and shows (*L*, *N*) induction of Wg (green) and eye suppression phenotype as marked by reduced Elav expression (red) in the eye discs. (*O*) Frequency analysis of the two eye suppression phenotypes of reduced eye and No-eye in adults when SATB1 is misexpressed (*P*–*U*) Conserved expression of Dve protein seen in *Lucilia sericata*, commonly known as blowfly, which exhibits sexual dimorphism in placement of eyes on head. (*P*, *Q*) The distance between the two eyes, separated by head cuticle, are shown (by double-sided arrow) in (*P*) male and (*Q*) female head. Note that the female eyes are more widely spaced than the males. (*R*, *S*) The eye imaginal disc from (*R*) male and (*S*) female larvae show a broader Dve expression domain in the antenna and dorsal head vertex region marked by white dotted line in female as compared to males. (*T*, *U*) Dve (red) expression is also highly conserved in the (*T*) wing and the (*U*) leg imaginal disc. (***P < 0.001; **P < 0.01; *P < 0.05).

In the developing eye imaginal disc, *wg,* a negative regulator of eye development (4), plays an important role in regulating MF progression, thereby limiting the size of the eye versus head domains (4, 13). To further investigate its mechanism of action, we tested whether *dve* can regulate Wg signaling to regulate the eye versus head fate decision. We hypothesize that if *wg* acts downstream of *dve*, then blocking Wg signaling in *dve*-expressing cells should exhibit dorsal eye enlargements and mimic the *dve* LOF phenotype. Consistent with this hypothesis, misexpression of *sgg* or *dTCF^DN^*^5^, Wg antagonists in the *dve* expression domain (*dve* > *sgg* and *dve* > *dTCF^DN5^*), resulted in a nonautonomous phenotype of dramatic eye enlargements, where the entire head of the fly transforms into eyes such that even the head cuticle as well as antennae are transformed into eyes (Fig. 5 *B* and *C*). Other nonautonomous phenotypes include dorsal eye enlargements extending into the ocelli of the head (*dve* > *sgg*, Fig. 5*D*). In the eye disc, in addition to dorsal eye enlargements, which are caused by head-to-eye-specific fate change in the dorsal head vertex region, we observed nonautonomous phenotypes where an ectopic MF, which presents like a mirror image of the normal MF, is formed. This validates the hypothesis that the cells normally fated to form the head changed to a de novo eye fate, as confirmed by expression of a panneuronal fate marker Elav (*dve* > *sgg*, Fig. 5*E*). In rare cases, three different eye fields were observed in the same disc, which include the i) endogenous eye field, ii) ectopic eye on the dorsal head vertex, and iii) antenna to eye fate transformation (*dve* > *dTCF^DN5^*, Fig. 5*F*). Since the primordium, which later becomes the eye, antenna, and head, is initially specified to just the eye fate, changes in morphogen levels make the tissue susceptible to fate transformations and changes. Thus, blocking Wg signaling in the *dve* expression domain of the developing eye disc affects both the head and antennal fields and transforms them into ectopic eyes.

These phenotypes also suggest that besides canonical Wg signaling, *dve-*expressing cells may affect paracrine Wg signaling (*SI Appendix*, Fig. S4) in the developing eye imaginal disc as well. Therefore, we prevented transport of Wg protein from the *dve*-expressing cells by down-regulating *porcupine* (25) *(porc^RNAi^)*. The rationale was that *dve-*expressing cells are the site of Wg production from where Wg distribution/transport occurs to generate its morphogen gradient in the developing eye imaginal disc that leads to determination of the eye- versus head-specific fate. If the rationale is true, then blocking Wg transport from *dve*-expressing cells would result in a similar phenotype of dorsal eye enlargements as seen in *dve* LOF (Fig. 1 *G* and *H*). The dorsal eye enlargements were seen when Wg transport was blocked in *dve*-expressing cells by downregulation of *porc^RNAi^* (*dve* > *porc^RNAi^,* Fig. 5*G*), or by blocking endocytic transport using Rab5^DN^ (26) (*dve* > *rab5^DN^,* Fig. 5*H*), or by eliminating the Dve-expressing cells by *hippo* misexpression (27, 28) that triggers cell death (*dve* > *hpo,* Fig. 5*I*). Next, we tested the role of *dve* in regulating Wg signaling by killing these cells using *ricin* (29) (*dve* > *Gal80^ts^+ricin*) in short time windows (developmental time intervals) during development using the Gal80^ts^ TARGET strategy (30). These experiments revealed that *dve* function during the second larval instar of development (48 to 96 h) critically regulates Wg signaling (Fig. 5*J*). Thus, during second larval instar of development, DV patterning gene *dve* functions in determining the eye versus head fate by controlling Wg levels in the eye imaginal disc, which in turn regulates the interocular distance or placement of the eye on the head. In the developing eye, signals are under timely control by upstream gene regulators reiterating the importance of cross talk between patterning genes and signaling pathways (31, 32).

Next, to test whether *dve* function in regulation of the interocular distance is evolutionarily conserved, we took two approaches: First, we tested transgenic *Drosophila* that express SATB1, the human ortholog of *dve*. Misexpression of two independent SATB1 transgenic lines in the eye (*ey* > *SATB^1^1, ey* > *SATB^2^1*) results in eye suppression phenotype(s) similar to the GOF of *dve*, suggesting that these genes are functionally conserved during eye development (Fig. 5 *K* and *M*). GOF of *SATB1* exhibits a range of eye suppression phenotypes (Fig. 5*O*) along with Wg induction (Fig. 5 *L* and *N*).

Diffusible signaling molecules are recycled and reused in various developmental processes. The mechanism by which *dve* regulates Wg signal has fundamental morphological consequences of delineation of eye versus head boundary. A highly conserved *dve* sequence observed across the *Drosophila* species (*SI Appendix*, Figs. S5 and S6 and Table S1) led us to compare *dve* expression in the eye- and wing-imaginal discs in other *Drosophila* species. Notably, Dve expression is conserved in the dorsal head vertex region in eye discs and wing pouch region of wing discs from other *Drosophila* species along the evolutionary scale (*SI Appendix*, Fig. S5). To further investigate the *dve* expression profile, we tested expression of Dve in *Lucilia sericata*, a fly with a sexually dimorphic trait of the interocular distance (33, 34) between the eyes on the head. The interocular distance is larger in females as compared to males (Fig. 5 *P* and *Q*). In the eye imaginal discs from the sexed larvae, we found that Dve expression domain on the dorsal head vertex is broader in females as compared to males (Fig. 5 *R* and *S*). Thus, *dve* expression plays an important role in determining the eye versus head fate and interocular distance in the developing insect eyes. Additionally, Dve expression is also conserved in the wing and leg imaginal discs of *L. sericata* (Fig. 5 *T* and *U*). Furthermore, we found that similar to Homothorax (Hth) (35), *dve* expression is observed in the extended stalk of the stalk-eyed fly where the eyes are placed on laterally extended stalks. This study only showed that *dve* expression and sequence are highly conserved in stalk-eyed flies (36). Loss of Wnt signaling has been reported to cause hypertelorism, which affects the interocular distance in patients and implicated in defective craniofacial development (6, 37). Changes in the levels of morphogens like Wnt/Wg are known to impact basic cellular processes like patterning, proliferation, and growth. During organogenesis, WNT signaling gradient plays vital roles in deciding the placement of internal organs in the body cavity; any deviation results in situs inversus where body parts are mislocalized such as the heart is on the right side (38–40). Therefore, such changes in signaling levels can directly impact the interocular distance during eye development and can exhibit phenotypes similar to orbital hypertelorism. Orbital hypertelorism is defined as increased distance between the eye orbits and can also result in abnormal placement of the eye (dystopia) (41, 42). This usually manifests with other developmental deformities of the eye and brain. Several studies have shown the importance of Wnt/Wg gradient and signaling during eye and craniofacial development (6, 41). Signaling and movement of morphogens have significant implications in establishing basic patterning (5, 43), facial morphology, and can cause developmental defects like hypertelorism, facial defects, cleft palate, etc. (6, 37, 44).

In our study, we show that a transcription factor, Dve, the *Drosophila* ortholog of human SATB1, has a conserved function in regulating the transcription of *wg* and thereby regulating the Wg morphogen gradient from a subset of cells in the developing eye–antennal imaginal disc to determine the placement of eyes on the head. Even though *pnr* acts upstream of *dve* in dorsal gene hierarchy, the role of Dve to regulate the Wg morphogen gradient is unique to *dve.* This is because *pnr* LOF or blocking Wg signaling in Pnr expression domain only showed dorsal eye enlargement whereas blocking Wg signaling in Dve-expressing cells changes the entire eye–antennal field to an eye (Fig. 5). In a study in humans, 42 individuals with a rare pathogenic variant in the SATB1 gene (MIM:602075; Gen Bank: NM001131010.4) were reported to show phenotypes of neurodevelopmental delay (97%), epilepsy (61%), facial dysmorphism (67%), and vision defects (55%). Furthermore, a patient with SATB1 E413K missense mutation showed hypertelorism along with other facial dysmorphic features (45). Our results also provide evidence for the role that SATB1 ortholog Dve plays in determining the variation in the interocular distance among different species by regulating *wg*. These studies using Dve/SATB1 uncover a regulatory mechanism that controls interocular distance during craniofacial development by regulating morphogen signaling by a transcription factor. Future studies are warranted to further dissect out other complex interactions during eye development and growth.

## Methods

### Fly Stocks.

The stocks used in this study are listed in FlyBase (http://flybase.bio.indiana.edu). The fly stocks are *ey*-Gal4 (23), *bifid (bi)*-Gal4 (12, 46), UAS-*dve*(16), UAS-*dve-IR dve^1^*(16, 47), FRT42D *dve^1^*(48), UAS-*pnr^D4^* (49), UAS-*pnr^ENR^* (50), y, w; FRT82B *pnr^vx6^*/CyO (46), UAS-*wg* (51), UAS-*sgg^S9A^*, UAS-*dTCF^DN^*(52), *ey*FLP; FRT82B Ubi-GFP, UAS-*porc^RNAi^* (25), UAS-*Rab5^DN^* (26), UAS-*hpo* (27, 28), UAS-*ricin* (29), UAS-*SATB^1^1,* UAS-*SATB^2^1*, *dve*Gal4*GFP*/CyO; *Gal80^ts^*/Tb, and *wg-lacZ/CyO* (23), a *lacZ* reporter which serves as the functional readout of the Wg signaling pathway. We used the Gal4/UAS system for targeted misexpression studies (53). All Gal4/UAS crosses were maintained at 25/29 °C. The adult flies from crosses were maintained at 25 °C, while the cultures after egg laying (progeny) were transferred to 25/29 °C for further growth (54, 55). The *ey*-Gal4 driver line directs expression of responder transgenes in the differentiating retinal precursor cells of the developing *Drosophila* eye imaginal disc. The *bi*-Gal4 directs expression in the dorsal and ventral eye and along the A/P axis of the wing. Other organisms used in this study include *Drosophila* species such as *D. melanogaster*, *D. yakuba*, *D. ananassae*, *D. virilis*, *D. pseudoobscura*, and *D. willistoni* and blow fly, *L. sericata*.

### Genetic Crosses.

The Gal4/UAS system was used to misexpress the gene of interest (53). All crosses were maintained at 18 °C, 25 °C, and 29 °C, unless specified, to sample different induction levels. A temperature-sensitive Gal80^ts^ system (30) was used to restrict the expression of a UAS-transgene of interest spatially and temporally.

### Genetic Mosaic Analysis.

The FLP/FRT system of mitotic recombination (19) was used to generate LOF clones of *pnr* in the eye. *eyFLP; FRT82B Ubi-GFP* females were crossed to *y, w; FRT82B pnr^vx6^* males. LOF clones (56) of *dve* in the eye were generated using a cell-lethal approach by crossing virgins of *eyFlp; FRT42D, cl-w^+^/CyO-GFP* to males of *FRT42D dve^1^*. GOF clones of *pnr* and *dve* were generated using the hs-FLP method by crossing *y, w, hsFLP122; P(Act > y^+^ > Gal4) 25 P(UAS-GFPS65T)/CyO* (57) flies to UAS-*pnr^D4^* or UAS-*dve* flies, respectively, and heat shock at 37 °C.

### Immunohistochemistry.

Eye–antennal imaginal discs were dissected from wandering third-instar larvae in 1× PBS and stained following standard protocol (54, 58). Primary antibodies used were rabbit anti-β-GAL (1:100; Cappel); rat anti-Elav (1:100), mouse anti-Wg (1:100; Developmental Studies Hybridoma Bank, DSHB), mouse anti-Disc large (1:100; DSHB); rabbit anti-Ey (1:200, a gift from Uwe Walldorf and Patrick Callaerts), mouse anti-Eya (1:100; DSHB), mouse anti-Dac (1:100; DSHB), and anti-Rabbit Dve (1:200). Secondary antibodies (Jackson Laboratories) used consisted of donkey anti-mouse IgG conjugated with Cy3 (1:250), donkey anti-rabbit IgG conjugated to Cy3 (1:250), and goat anti-rat IgG conjugated with Cy5 (1:200). The tissues were mounted in Vectashield (Vector Labs), and all the immunofluorescence images were captured using the Olympus FluoView 1000 Laser Scanning Confocal Microscope.

### Western Blot.

Protein samples were prepared using the adult *Drosophila* heads from wild-type, *ey*-Gal4, *ey*-Gal4 > UAS-*dve*, and *ey*-Gal4 > UAS *dve-IR dve^1^* following the standardized protocol (59, 60). The protein samples were centrifuged, and the supernatant was collected and quantified using Nanodrop. The samples were normalized and loaded in wells of a 10% Sodium dodecyl sulfate polyacrylamide gel electrophoresis (SDS-PAGE) gel. The primary antibodies used were anti-mouse Wg (1:1,000, 4D4, DSHB) and anti-mouse tubulin (1:12,000, Sigma-Aldrich Corp.), and secondary antibodies horseradish peroxidase–conjugated anti-Mouse IgG-HRP (1:5,000, Santa Cruz Biotechnology). The signal was detected using the supersignal chemiluminescence substrate kit (Pierce Biotechnology, ThermoFisher Scientific, Rockford, IL USA), and the images were captured using the LI-COR Odyssey^®^ XF.

### Real-Time qPCR (RT-qPCR).

*Drosophila* eye imaginal discs (n = 80) for *ey*-Gal4 and *ey* > *dve* were collected and homogenized in TRIzol Reagent (Invitrogen, Cat# 15596026) to extract the total RNA (61). The quality and quantity of the RNA were determined using the Nanodrop 2000 spectrophotometer (Thermo Scientific). Using the first-strand cDNA synthesis kit (GE Healthcare, Cat# 27926101), 1 µg of total RNA was used for cDNA synthesis. Real-time qPCR was performed using Bio-Rad iQ SYBR Green Supermix (Bio-Rad, Cat# 1708860) according to the standard protocol (61, 62). Fold change was calculated using the comparative ΔΔCT method (fold change = 2^−ΔΔCT^). All samples were run in triplicates (n = 3). Statistical significance was determined at 95% confidence (*P* < 0.05) using Student’s *t* test.

Primers:

GAPDHFw5′-GGCGGATAAAGTAAATGTGTGC-3′
GAPDHRev5′-AGCTCCTCGTAGACGAACAT-3′
wgFw5′-TCAGGGACGCAAGCATAATAG-3′
wgRev5′-CGAAGGCTCCAGATAGACAAG-3′

## Supplementary Material

Appendix 01 (PDF)

## Data Availability

All study data are included in the article and/or *SI Appendix*.
