# Supplementary material for: Genetic mechanism regulating diversity in the placement of eyes on the head of animals
Source: Proc Natl Acad Sci U S A. 2024 Apr 8;121(16):e2316244121. doi: 10.1073/pnas.2316244121 (PMC11032433; doi:10.1073/pnas.2316244121)
Supplement: Supplementary file 1 — Appendix 01 (PDF) [file pnas.2316244121.sapp.pdf]

Supplementary Data

Supplementary Figure 1 (S1)

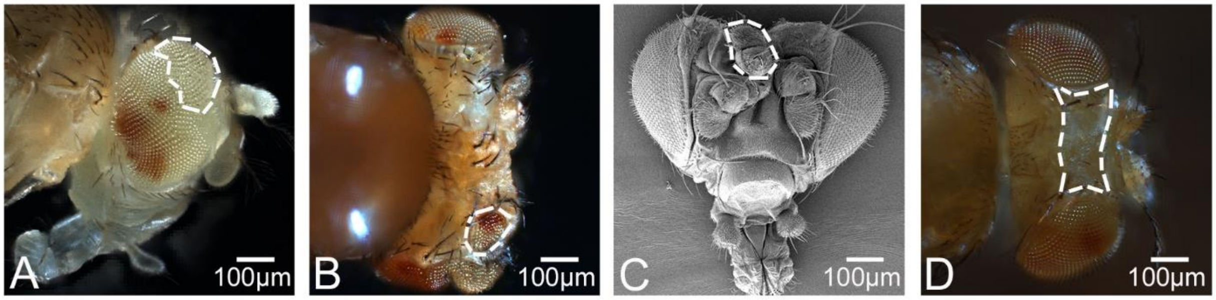

Dorsal eye enlargement	Ectopic eyes	Antennal duplications	Ocelli loss
20/245	5/245	20/245	51/245
0.0816	0.0204	0.0816	0.208

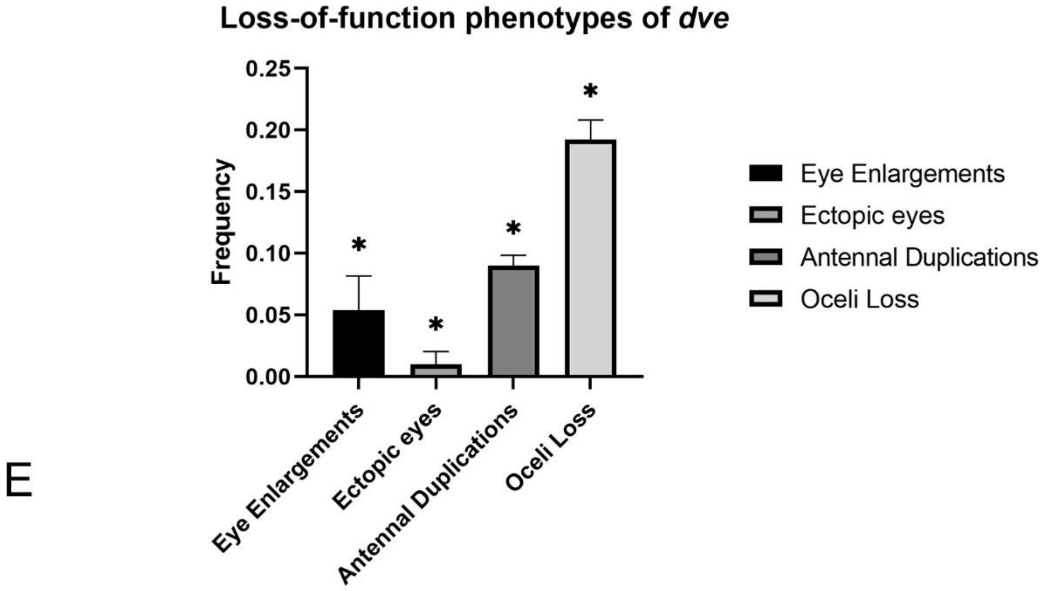

Supplementary Figure 1: Loss-of-function phenotypes of *dve* in the *Drosophila* eye. Loss-of-function of *dve* results in (A) dorsal eye enlargements (8.16%), (B) ectopic eyes in the dorsal head cuticle region (2.04%, white outline), (C) a mirror image duplication of the antenna (8.16%, white outline) and (D) loss of ocelli in the adult fly (20%). (E) Table showing the frequency of mutant phenotypes and graph showing the frequency of these phenotypes.

Supplementary Figure 2 (S2)

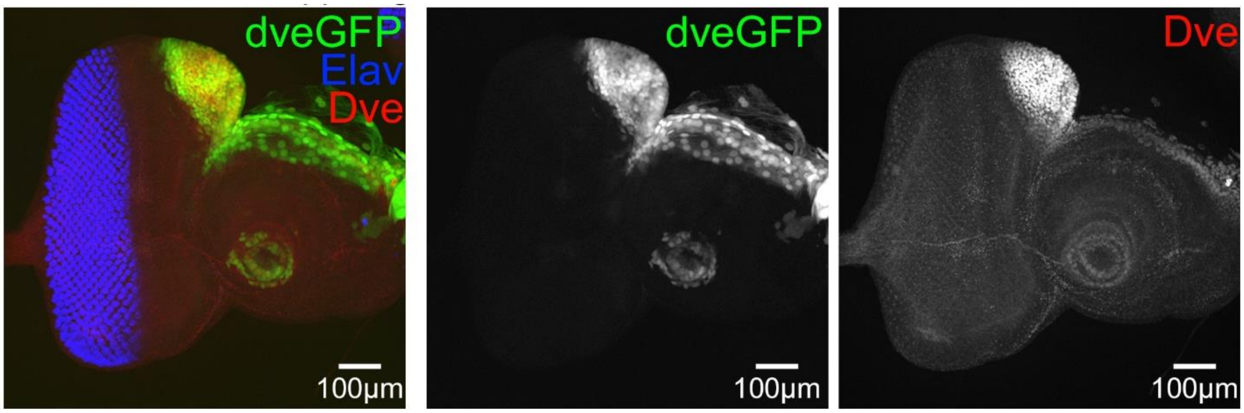

Supplementary Figure 2: *dve* Gal4 drive GFP reporter expression mimics Dve protein localization in the developing eye disc. (A-A”) *dve*-Gal4 drives expression of GFP (*dve* Gal4>UAS-GFP, *dve*>GFP) reporter in the dorsal head vertex of the developing third instar eye imaginal disc. Note that (A’) *dve*>GFP expression has near complete overlap with (A”) Dve protein localization in the dorsal head vertex region.

Supplementary Figure 3 (S3)

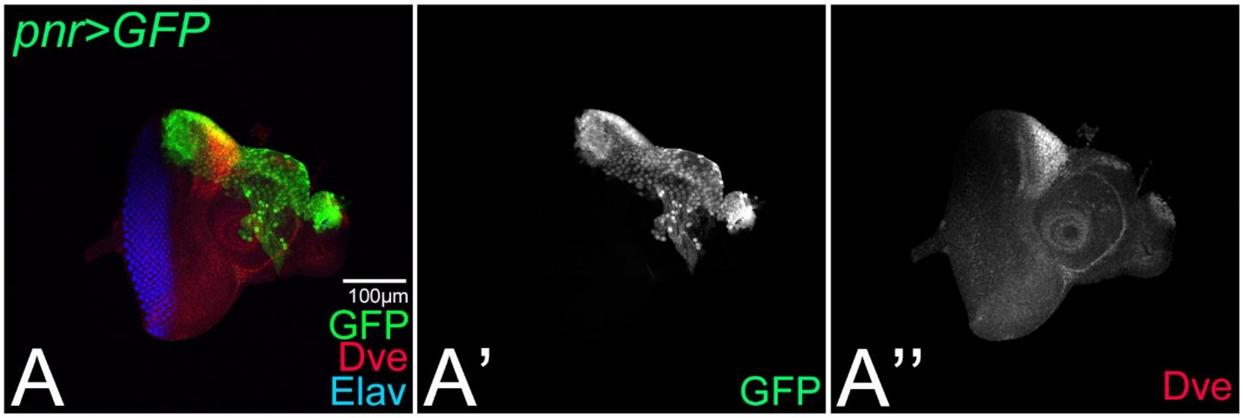

Supplementary Figure 3 (S3): Dorsal eye fate selector *pannier (pnr)* expression (*pnr>GFP*) exhibits partial overlap with *dve* in the head vertex region of developing eye imaginal disc. (A-A”) *pnr*-Gal4 drives expression of GFP reporter (*pnr*-Gal4>UAS-GFP, *pnr*>GFP) in the peripodial membrane and dorsal margin of developing third instar eye imaginal disc whereas Dve is localized in the dorsal head vertex region of the disc proper. Note that (A’) *pnr>GFP* expression has minimal overlap with (A”) Dve protein localization in the dorsal head**.**

Supplementary Figure 4 (S4)

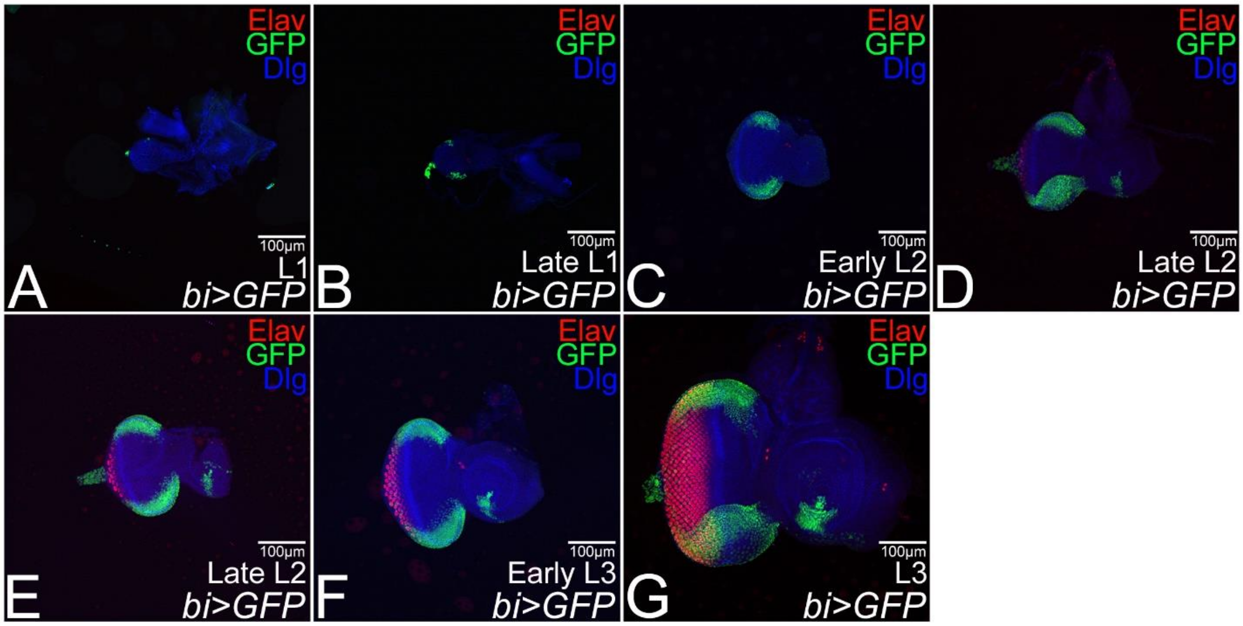

Supplementary Figure 4: *bi*-Gal4 drives expression of GFP reporter in the developing eye-antennal imaginal disc. bi-Gal4 drives expression of GFP reporter on the dorsal (D) and ventral (V) margins of the eye-antennal imaginal discs of larvae at (A) First instar stage, (B) Late first-instar, (C) Early second- instar, (D, E) Late second- instar, (F) Early third- instar, and (G) Late third instar stage.

Supplementary Figure 5 (S5)

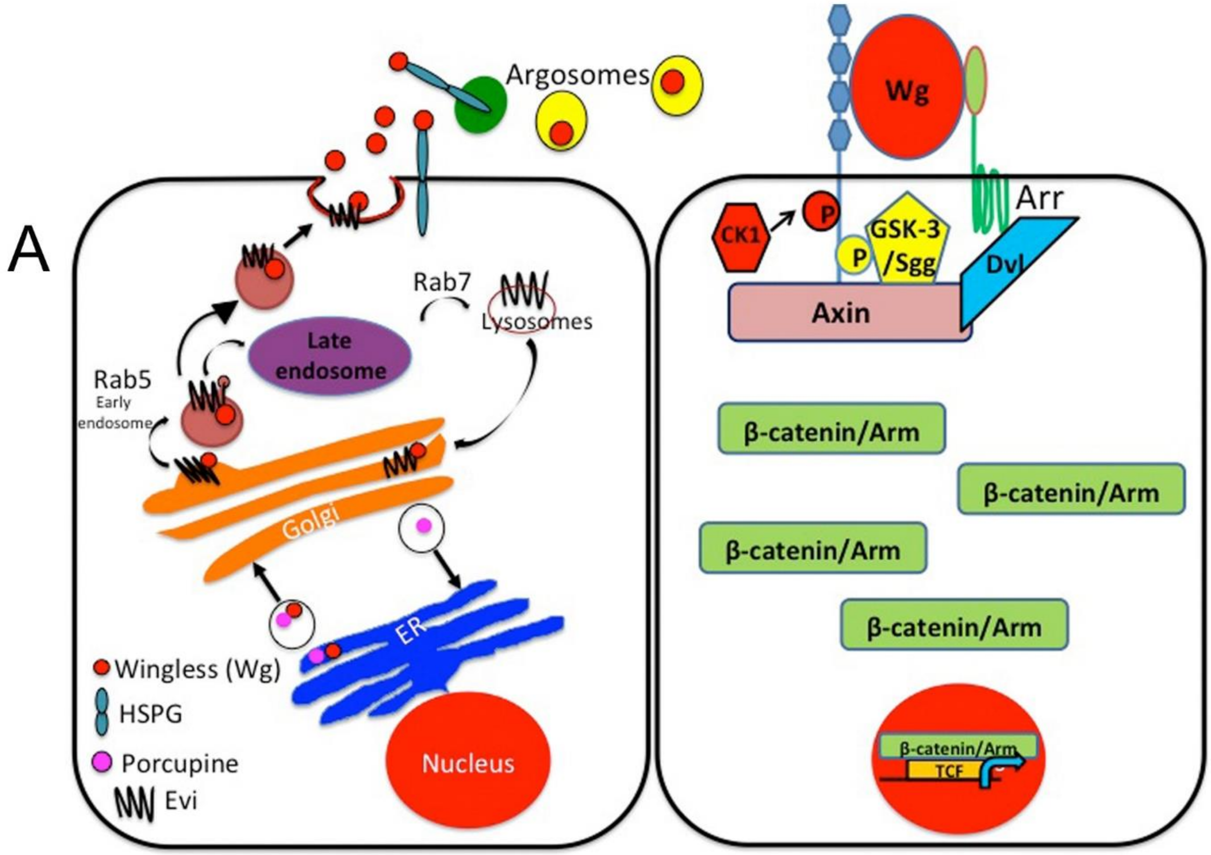

Supplementary Figure 5: **Schematic representation of Wg signaling.**

Supplementary Figure 6 (S6)

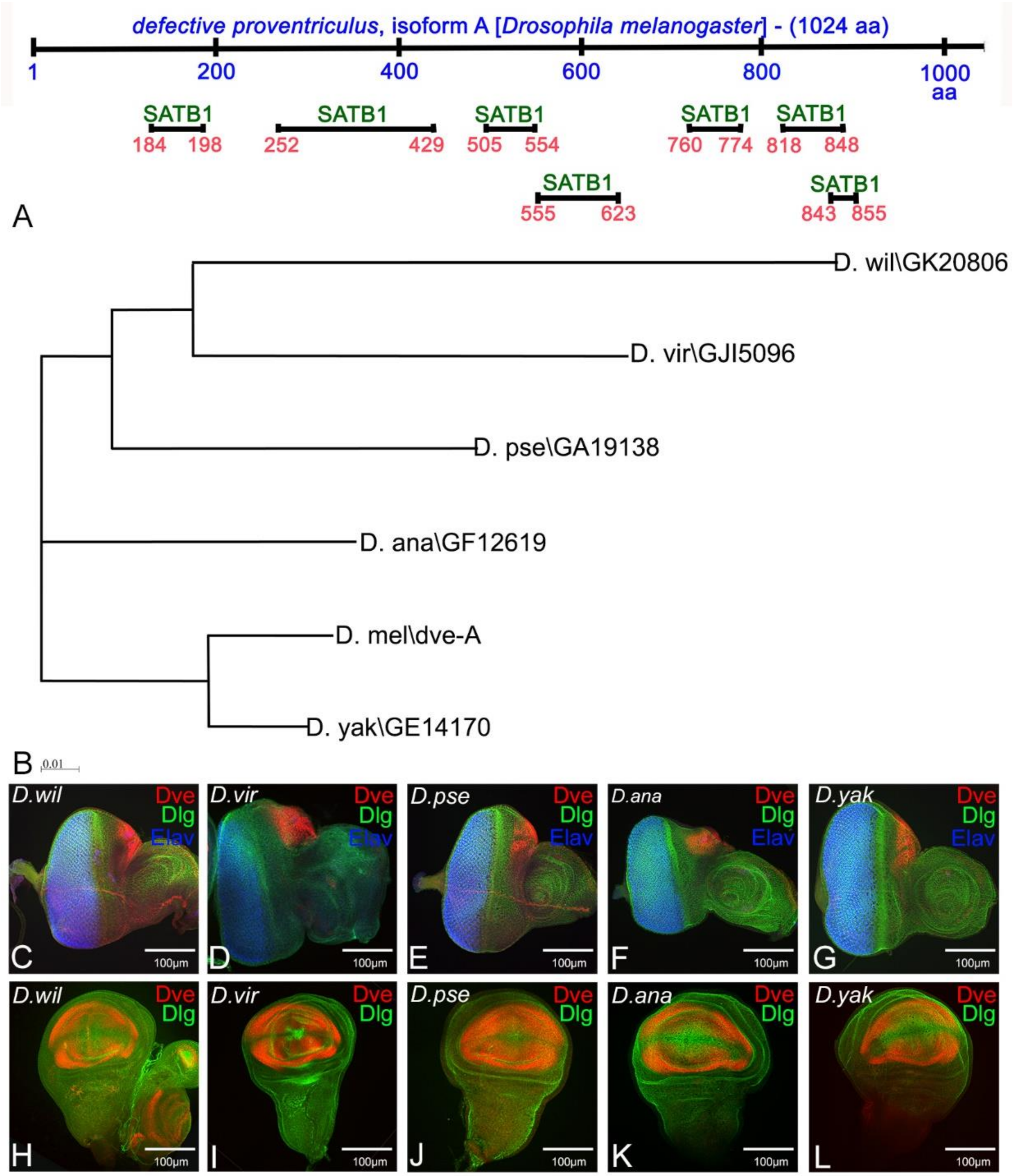

Supplementary Figure 6: Phylogenetic tree and expression of *dve* in different *Drosophila* species. (A) The phylogenetic tree was generated after alignment of the *dve*-transcript A by CLUSTAL using the Tree view software. Dve expression pattern (green) in (B-F) the eye-antennal and (G-K) wing imaginal disc of (B, G) *D. willistoni*, (C, H) *D.virilis*, (D, I) *D. pseudoobscura*, (E, J) *D. ananassae*, and (F, K) *D. yakuba*. Note that the Dve expression in eye disc of all the species

is restricted to dorsal head vertex and is conserved. However, (G-K) Dve expression in wing imaginal disc varies in robustness in the wing pouch area.

Supplementary Figure 7 (S7)

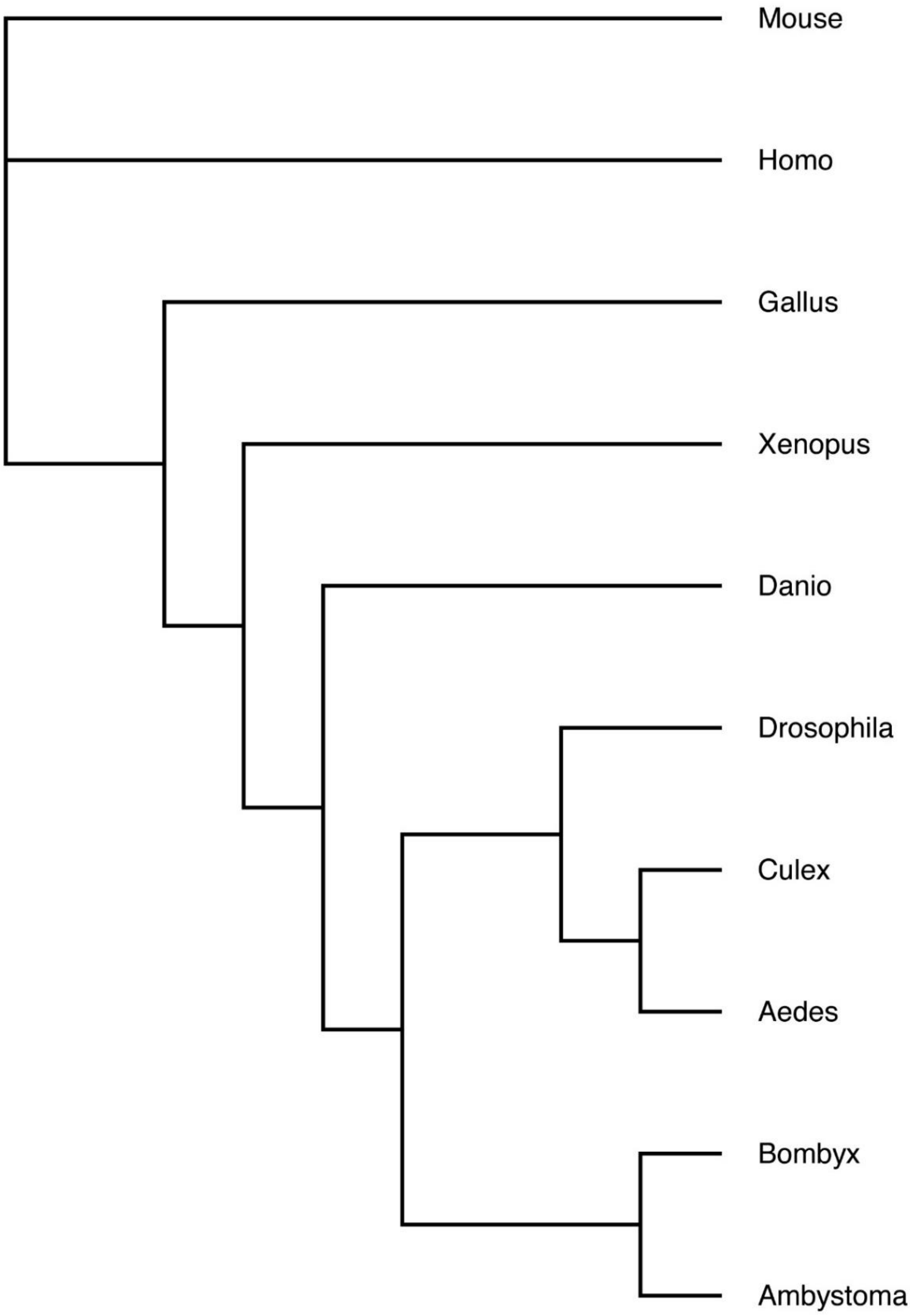

Supplementary Figure 7: Clustal analysis shows the homology of *dve* with other homologous / orthologous sequences of *dve*/ SATB1 in various organisms.

Supplementary Table 1: **Conservation of *dve* among organisms that share a conserved homologous sequence.**

SeqA	Name	Length	SeqB	Name	Length	Score
1	dve-PA-Drosophila	3075	2	Culex	2115	70.07
1	dve-PA-Drosophila	3075	3	Aedes	1986	70.69
1	dve-PA-Drosophila	3075	4	Bombyx	2278	62.29
1	dve-PA-Drosophila	3075	5	Human	2292	57.24
1	dve-PA-Drosophila	3075	6	Mouse	2295	58.91
1	dve-PA-Drosophila	3075	7	Danio	2448	54.98
1	dve-PA-Drosophila	3075	8	Gallus	3429	49.98
1	dve-PA-Drosophila	3075	9	Chicken	2196	58.47
1	dve-PA-Drosophila	3075	10	Xenopus	2819	49.59
2	Culex	2115	3	Aedes	1986	81.57
2	Culex	2115	4	Bombyx	2278	59.86
2	Culex	2115	5	Human	2292	52.77
2	Culex	2115	6	Mouse	2295	52.39
2	Culex	2115	7	Danio	2448	51.82
2	Culex	2115	8	Gallus	3429	59.15
2	Culex	2115	9	Chicken	2196	49.83
2	Culex	2115	10	Xenopus	2819	55.79
3	Aedes	1986	4	Bombyx	2278	60.27
3	Aedes	1986	5	Human	2292	54.93
3	Aedes	1986	6	Mouse	2295	53.27
3	Aedes	1986	7	Danio	2448	55.99
3	Aedes	1986	8	Gallus	3429	62.79
3	Aedes	1986	9	Chicken	2196	53.88
3	Aedes	1986	10	Xenopus	2819	57.05
4	Bombyx	2278	5	Human	2292	51.45
4	Bombyx	2278	6	Mouse	2295	50.75
4	Bombyx	2278	7	Danio	2448	51.49
4	Bombyx	2278	8	Gallus	3429	59.83
4	Bombyx	2278	9	Chicken	2196	50.41
4	Bombyx	2278	10	Xenopus	2819	54.39
5	Human	2292	6	Mouse	2295	91.49
5	Human	2292	7	Danio	2448	62.43
5	Human	2292	8	Gallus	3429	87.74
5	Human	2292	9	Chicken	2196	66.8
5	Human	2292	10	Xenopus	2819	79.32
6	Mouse	2295	7	Danio	2448	62.22

Supplementary Table 2: Genetic crosses used in this study

No.	Genotypes
1	*y, w*; *ey*-Gal4/ CyO X *y, w*; Sco/Cyo; UAS-*dve*/ *TM3 Sb e Ser*
2	y w; *ey*-Gal4 (III) X *y, w;* UAS-SATB1/*CyO*
3	y w; *ey*-Gal4 (III) X y, w; UAS-SATB2/CyO
4	*y w; ey*-Gal4 (III) X *y, w; wg-lacZ/CyO*; UAS-*dve*/ *TM3SB e Ser*
5	*y w; ey*-Gal4 (III) X *y, w,* UAS-*dve*^{RNAi} ,*dve* ^{1}
6	*y, w, ey* FLP; FRT42D cl -w^{+}/CyO-GFP *X* *y, w;* FRT42D, *dve*^{1}/CyO
7	*y w, hsFLP*^{122}; Act>y+> Gal4, UAS-GFP/CyO X *y,w;* UAS-*dve/ TM3 Sb e Ser*
8	*y, w, ey* FLP; FRT82B ubi-GFP/TM6B Tb *X* *y, w;* FRT82B, *pnr*^{vx6}/*TM6BTb*
9	*y, w, bi*-Gal4/FM7 X *y, w,* UAS-pnr^{ENR20}/CyO
10	*y, w, bi*-Gal4/FM7 X *y, w,* UAS-*pnr*^{D4}/*TM3 Sb e Ser*
11	*y, w, bi*-Gal4/FM7 X *y, w;* UAS-*dve*/*TM3 Sb e Ser*
12	*y, w, bi*-Gal4/FM7 X *y, w,* UAS-GFP/CyO
13	*y, w, bi*-Gal4/FM7 X *y, w,* UAS-*ey* /CyO
14	*y, w, bi*-Gal4/FM7 X *y, w,* UAS-*wg*/CyO
15	*y, w, dve*-Gal4/ CyO X *y, w,* UAS-GFP/CyO
16	*y, w, dve*-Gal4, UAS-GFP/ CyO X ,*y w,* UAS-ey/ CyO
17	*y, w, dve*-Gal4, UAS-GFP/ CyO X *y, w,* UAS-*flu Δ arm/CyO*
18	*y, w, dve*-Gal4, UAS-GFP/ CyO X *y, w,* UAS-*porc* ^{RNAi}/*CyO*
19	*y, w, dve*-Gal4, UAS-GFP/ CyO X *y, w,* UAS-*sgg*^{A9}/*CyO*
20	*y, w, dve*-Gal4, UAS-GFP/ CyO X *y, w,* UAS-*dTCF*^{DN5}/ *TM3 Sb e Ser*
21	*y, w, dve*-Gal4, UAS-GFP/ CyO X *y, w,* UAS-*rab*^{5DN}/*CyO*
22	*y, w, dve*-Gal4, UAS-GFP/ CyO X *y, w,* UAS-*mst*^{8}/*CyO*
